# Trends of pre-hospital emergency medical services activity over 10 years: a population-based registry analysis

**DOI:** 10.1186/1472-6963-14-380

**Published:** 2014-09-10

**Authors:** Valérie Pittet, Bernard Burnand, Bertrand Yersin, Pierre-Nicolas Carron

**Affiliations:** Institute of social & preventive medicine (IUMSP), Lausanne University Hospital, Route de la Corniche 10, CH-1010 Lausanne, Switzerland; Emergency Service, Lausanne University Hospital, Lausanne, Switzerland

**Keywords:** Pre-hospital emergency medical services, Epidemiology, Health Services, Public Health, Registers, Activity indicators

## Abstract

**Background:**

The number of requests to pre-hospital emergency medical services (PEMS) has increased in Europe over the last 20 years, but epidemiology of PEMS interventions has little be investigated. The aim of this analysis was to describe time trends of PEMS activity in a region of western Switzerland.

**Methods:**

Use of data routinely and prospectively collected for PEMS intervention in the Canton of Vaud, Switzerland, from 2001 to 2010. This Swiss Canton comprises approximately 10% of the whole Swiss population.

**Results:**

We observed a 40% increase in the number of requests to PEMS between 2001 and 2010. The overall rate of requests was 35/1000 inhabitants for ambulance services and 10/1000 for medical interventions (SMUR), with the highest rate among people aged ≥ 80. Most frequent reasons for the intervention were related to medical problems, predominantly unconsciousness, chest pain respiratory distress, or cardiac arrest, whereas severe trauma interventions decreased over time. Overall, 89% were alive after 48 h. The survival rate after 48 h increased regularly for cardiac arrest or myocardial infarction.

**Conclusion:**

Routine prospective data collection of prehospital emergency interventions and monitoring of activity was feasible over time. The results we found add to the understanding of determinants of PEMS use and need to be considered to plan use of emergency health services in the near future. More comprehensive analysis of the quality of services and patient safety supported by indicators are also required, which might help to develop prehospital emergency services and new processes of care.

## Background

Pre-hospital emergency medical services (PEMS) were historically established in European countries in the early 1980 (Germany, Spain, Scandinavia), based on previous experiences in Belfast [1], the USA [2] and France [3]. One main difference between US and European PEMS is the presence of physicians in most European PEMS, impacting therefore on ambulance system organization and on-site medical strategy. Detailed activities of PEMS were previously described [4–9]. Usually, the descriptive characteristics of PEMS organisations imply operational indicators. Clinical parameters and treatment options are proposed to better describe PEMS activity, of whom patient immediate outcomes [10]. However, benchmarking of quality indicators are not easy to achieve, due to the multitude of PEMS organisations all over the world [11–13]. The number of validated indicators is limited, mainly focusing on specific pathologies, but not on system-wide process evaluation [14–16]. Some indicators are nevertheless useful and could be easily documented throughout the prehospital patient pathway, thus allowing external benchmarking [17, 18].

The number of requests to PEMS has increased in Europe over the last 20 years [19, 20]. A three-fold increase in the number of calls to PEMS was observed in Paris over 10 years [3], with a two-fold increase in hospital emergency admissions, inducing longer waiting times in both services. Among reasons given to explain this trend are the growth and ageing of population, a limited access to primary care physicians, and a wider public awareness of specific health problems (e.g. implementation of a single emergency call number, campaigns on stroke or acute myocardial infarction). Moreover, the hypothesis that the observed trend might be due to a parallel increase in the number of inappropriate calls was not confirmed, showing therefore a limited misuse of the system [21]. Little is known about the epidemiology and evolution of a European PEMS over more than 10 years. Only limited data on specific medical topics or only some parts of the system were investigated [22, 23]. There is however an important medical, policy, and public health interest to analyze the activity and trends of the whole PEMS concept, including the emergency call center, the pre-hospital emergency ambulances and physicians’ response, as well as the admission of the patients into the hospital emergency network [22].

The main aim of this analysis was to describe the time trends of all requests to PEMS in a region of western Switzerland from 2001 to 2010. Secondary objectives were to describe PEMS interventions, and to analyse trends of a selection of processes and results indicators over time to characterize PEMS activity.

## Methods

### Study design and population

This study was based on data routinely and prospectively collected for each PEMS request in the Canton of Vaud, Switzerland, from January 2001 to December 2010. This Swiss Canton (Canton de Vaud) is located in the western French-speaking part of Switzerland, has an area of 3′212 km2, and a population that has grown from 621′784 to 708′177 between 2001 and 2010, representing approximately 10% of the whole Swiss population.

### Description of the state PEMS

Since 2001, the PEMS include a unique emergency call center (ECC), 23 emergency ambulances, a rescue helicopter and 8 physician-manned emergency resuscitation vehicles. The ECC is staffed by trained nurses or paramedics, using a specific keyword-based dispatch protocol. Ambulances are staffed with fully trained paramedics and constitute the initial response of the PEMS. Primary interventions include on-site and at-home emergencies, whereas secondary interventions include urgent inter-hospital transfers. Primary interventions are classified by the ECC in three priority categories, according to the potential severity of the situation: P1 (life-threatening emergencies, requiring immediate ambulance response with use of lights and sirens), P2 (health-threatening situations, using light and sirens if necessary) and P3 (non-emergency situations which nevertheless require an ambulance intervention and a transport to a hospital). Pre-hospital emergency physicians may be sent by ECC simultaneously on site by ground (8 emergency resuscitation vehicles, called SMUR) or by air (one rescue helicopter). They are engaged specifically in the case of cardiac arrest, major trauma, respiratory distress, coma or other life-threatening emergencies, or secondary at the request of the ambulance’s paramedics on site. Emergency physicians’ interventions may be cancelled by paramedics when they arrived first on site and faced a non-emergency situation.

Patients are transported to one of the seven regional hospitals of the Canton, or the University Hospital of Lausanne (CHUV), depending on the severity of the pathologies and the proximity of the hospital. The CHUV, a 1500-bed university hospital located in Lausanne, is considered the primary hospital for its immediate catchment area, but it also serves as the Level 1 Trauma and Burn center and tertiary reference hospital for the Canton.

### Databases and variables

Any request to PEMS is made through the 144 telephone number of the ECC, in charge of PEMS resources management, using an electronic decision-support system (DSS). This system is based on algorithms taking into account the types and reasons of the interventions (e.g., road accident, fall, medical problem), and of a list of keywords describing the patient’s clinical situation (e.g., coma, dyspnoea, chest pain, haemorrhage). For each emergency call, a record is automatically created in the DSS database. Among the total number of calls, some could be withdrawn by the rescuers immediately or once on the scene (e.g., in the case of a person who finally left the scene, or who was told unconscious instead of asleep). An identification number is assigned for actual interventions and a set of regulation data are collected. It comprises characteristics of the caller, chronological data, type of situation and keywords, potential severity of the patient’s situation according to the NACA score [24], PEMS resources engaged, patient’s name, age, and gender. Each resource engaged (ambulance, emergency physician, rescue helicopter) completes a medical report, from which data was recorded afterwards in distinct resource-specific databases. Ambulance reports comprise regulation data, evaluation of the patient on site (severity, life-saving measures), and action undertaken (e.g. hospital transport, call of the SMUR, person not conveyed to the hospital, or death on site). SMUR and helicopter reports contain the chronology of the intervention and eventual reasons for delays (entrapment), regulation data, life-saving measures, treatments and procedures on site, transport indications and immediate outcome of the patient at time of hospital admission; the confirmed diagnosis and immediate outcome are prospectively collected after 48 hours. The recorded data is in agreement with the Utstein recommendations for uniform reporting of cardiac arrest, major trauma and with the Uniform PEMS Data Conference [25–28]. Each consecutive month, a complete PEMS dataset of each intervention is entered by a unique data manager in a central data registry, with a copy provided to the Institute of social and preventive medicine (IUMSP). IUMSP is in charge of performing the descriptive analyses ordered by the canton, and presented in this study.

Variables were patient’s gender and age (<16, 17–49, 50–79, > = 80), types of intervention (primary or secondary), priority level (P1, P2 or P3), category of phone appellant (family, bystander, physician, nurse, ambulance, patient, police, fireman), NACA score (1 to 7), types and reasons of the intervention (accident, fight, medical problem, fire or toxic spill, other), ECC keywords, place of intervention (home, public place, school or workplace, hospital or private practice, sport area, other). Duration and delays were recorded for ambulance, and for SMUR interventions. For SMUR interventions, additional medical data were also recorded, comprising the final diagnosis and outcome at 48 hours. Process and system-related quality indicators were defined according to the proposed definitions of the Joint Commission on Accreditation of Healthcare Organisation. They include time to start (duration in minutes from alarm to start of the vehicle), time to response (duration in minutes from alarm to arrival of the vehicle on scene) and duration on scene (duration in minutes from arrival to departure of vehicle from the scene). Survival to hospital admission and at 48 hours after admission was calculated per year for all documented interventions and at 48 hours after admission for interventions related to specific medical diseases (cardiac arrest, myocardial infarction, stroke, pulmonary embolism, asthma, COPD, sepsis).

### Statistical analysis

Descriptive cross-tables with number and rates or percentages were obtained for all variables according to years when PEMS interventions were performed. We assumed independency for all, even if it was not possible to guarantee this assumption because working on an anomymised database. Patients could have used PEMS more than once. However, we hypothesized that, due to the low frequency of these situations among the large number of cases included, this had a negligible impact on the results. All analyses were performed with STATA 12.1.

### Ethical aspects

The copy of the central data registry provided by the canton to the Institute of social and preventive medicine (IUMSP) is anonymous. The investigators were thus unaware of the identity of the persons for whom PEMS was engaged during the period of the study. The study protocol was submitted to the Ethical Committee of the University of Lausanne, Canton of Vaud, as well as to the Health Care authority of the canton, who both agreed to the study. As agreed by the Ethical Committee, this study was performed on anonymously collected or anonymised health-related data, therefore there was no need of written informed consent from individual patients (Federal Act on Research involving Human Beings, Art. 2).

## Results

### Description of PEMS interventions

**Figure 1:**
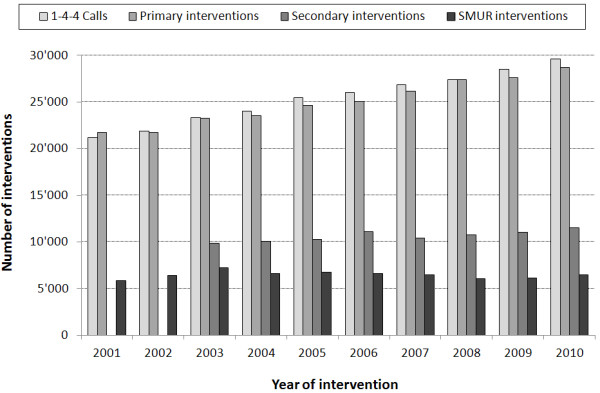
**Evolution of the number of calls to the 144 ECC and PEMS interventions between 2001 and 2010.** ECC: emergency call center; PEMS: prehospital emergency medical services; primary interventions = on-site and at-home emergencies; secondary interventions = urgent inter-hospital transfers (secondary interventions were not recorded in 2001 and 2002); SMUR: emergency physicians sent simultaneously onsite.

An increase (Figure 1) in the number of calls to the 144 ECC was observed between 2001 and 2010. A total of 21′160 calls were recorded in 2001, as compared to 29′593 ten years later, representing a 39.8% increase of calls. A similar evolution was observed for PEMS interventions during that period, with a rate per 1000 inhabitants increasing from 34 to 39 (Table 1). The highest increase was observed among patients aged 80–89 (171 per 1000 inhabitant in 2001 vs. 214 in 2010) and aged ≥ 90 (259 per 1000 inhabitants in 2001 vs. 391 in 2010). Nearly 40% of the population requesting an intervention was aged <50, a proportion slightly decreasing over time (40.1% in 2001 vs. 36.5% in 2010). Half of the interventions concerned males.

**Table 1:** **Characteristics of the population having requested PEMS intervention between 2001–2010 (numbers and percentages)**

Variables	2001	2002	2003	2004	2005	2006	2007	2008	2009	2010
**Primary interventions**	21722	21753	23225	23510	24630	25048	26170	27376	27603	28697
Nb/1000 inhabitants	34	34	36	35	37	37	38	39	38	39
**SMUR interventions**	5865	6431	7230	6634	6724	6611	6452	6024	6090	6453
Nb/1000 inhabitants	9	10	11	10	10	10	9	8	8	9
**Age (years)**										
<16	1394 (6.6)	1383 (6.5)	1322 (5.9)	1218 (5.4)	1274 (5.4)	1308 (5.4)	1353 (5.3)	1340 (5.1)	1635 (6.1)	1565 (5.6)
17-49	7108 (33.5)	7162 (33.8)	7591 (33.6)	7625 (33.5)	7828 (32.9)	7877 (32.5)	8293 (32.7)	8357 (31.5)	8475 (31.7)	8547 (30.9)
50-79	7635 (36.0)	7474 (35.2)	7650 (33.8)	7636 (33.9)	7994 (33.6)	8139 (33.4)	8571 (33.6)	9032 (34.0)	9303 (34.7)	9653 (34.6)
> = 80	5076 (23.9)	5179 (24.4)	6047 (26.7)	6281 (27.6)	6706 (28.2)	6972 (28.7)	7202 (28.3)	7807 (29.4)	7359 (27.5)	8039 (28.9)
**Male gender**	10460 (49.4)	10603 (50.0)	11344 (50.2)	11331 (49.8)	11540 (48.5)	12053 (49.6)	12522 (49.3)	13015 (49.1)	13317 (49.8)	13742 (49.4)
NACA Score**										
<4			18735 (82.8)	19220 (84.5)	19851 (83.4)	20344 (83.8)	21118 (83.1)	22229 (83.8)	22490 (84.0)	23469 (84.4)
4-6			3429 (15.2)	3108 (13.7)	3485 (14.6)	3487 (14.4)	3853 (15.2)	3853 (14.5)	3786 (14.1)	3877 (13.9)
7			470 (2.1)	437 (1.9)	468 (2.0)	465 (1.9)	451 (1.8)	457 (1.7)	498 (1.9)	460 (1.7)

PEMS: prehospital emergency medical services; SMUR: emergency physicians sent simultaneously onsite;

**The NACA Score is divided into 7 categories: NACA 0 (no injury), 1 (slight injury, no medical intervention required), 2 (light-to-moderately heavy injury, ambulatory medical clarification necessary), 3 (heavy, but not life-threatening injury or illness, stationary treatment necessary, and frequently also local emergency medical measures), 4 (heavy injury, for which the short-term development of a life threat cannot be excluded), 5 (acute lethal danger), 6 (resuscitation), 7 (death).

The intervention rate of pre-hospital emergency physicians (SMUR) varied between 9 and 11 per 1000 inhabitants, the highest rate seen among people aged 80–89 (38 per 1000 inhabitants in 2001 versus 43 in 2010) and ≥ 90 (52 per 1000 inhabitants in 2001 versus 59 in 2010). From 2001 to 2010, priority 1 interventions decreased from 75% to 50% of all ambulances interventions. Interventions requiring the presence of a physician increased between 2001 (N = 5865) and 2003 (N = 7230), denoting the setting up of the system. Most of the calls to the ECC were made during day-time (7 am-7 pm: 62%), without any difference according to the day of the week. In 2001, calls from bystanders/witnesses and physicians had a similar proportion (30%); over years however, physicians calls decreased to 13.4% in 2010.

### Medical aspects

All together, interventions were more frequently requested for medical problems (66% over the study period), especially for coma, chest pain, or respiratory distress (Table 2). 28% of the interventions concerned trauma, with a decreasing proportion of them requesting the SMUR (16.8% in 2001 vs. 11.9% in 2010); this was particularly observable for the paediatric population where SMUR interventions for trauma decreased to 23.2%. A progressive reduction in the proportion of road traffic accidents was observed between 2001 (n = 1353; 9%) and 2010 (n = 1414; 5%). Interventions for medical diseases increased over time, especially for those requesting the SMUR (71.1% in 2001 vs. 77.6% in 2010). Overall, more than half of the SMUR interventions were made for cardio-vascular pathologies and one quarter for respiratory or thrombo-embolic pathologies. In terms of potential severity, NACA scores for primary ambulance interventions (Table 1) remained constant in proportions over years; about one third were NACA 0–2, 53% NACA 3 and 14% NACA 4–6. At the same time, NACA 4–6 scores for SMUR interventions increased by 10% between 2001 and 2010.

**Table 2:** **Medical characteristics of the population having requested PEMS intervention between 2001–2010 (numbers and percentages)**

Variables	2001	2002	2003	2004	2005	2006	2007	2008	2009	2010
**Primary interventions**	21722	21753	23225	23510	24630	25048	26170	27376	27603	28697
Medical problem*										
Trauma	5962 (28.1)	6138 (28.9)	6599 (29.2)	6634 (29.1)	6806 (28.6)	6721 (27.7)	7261 (28.6)	7399 (27.9)	7661 (28.6)	7965 (28.6)
Medical problem										
*Coma*	1804 (8.5)	1902 (9.0)	1991 (8.8)	2028 (8.9)	2024 (8.5)	2169 (8.9)	2419 (9.5)	2437 (9.2)	2505 (9.4)	2548 (9.2)
*Chest pain*	1594 (7.5)	1610 (7.6)	1586 (7.0)	1659 (7.3)	1697 (7.1)	1847 (7.6)	1967 (7.7)	1865 (7.0)	1565 (5.9)	1715 (6.2)
*Dyspnoea*	1233 (5.8)	1300 (6.1)	1442 (6.4)	1314 (5.8)	1544 (6.5)	1633 (6.7)	1710 (6.7)	1699 (6.4)	1693 (6.3)	1613 (5.8)
*Cardiac arrest*	506 (2.4)	495 (2.3)	500 (2.2)	466 (2.1)	533 (2.2)	544 (2.2)	525 (2.1)	543 (2.1)	574 (2.1)	559 (2.0)
**Place of intervention**										
Home			13436 (59.4)	13719 (60.3)	14575 (61.2)	15207 (62.6)	15488 (60.9)	15949 (60.1)	15999 (59.6)	16904 (60.6)
Public place			6229 (27.5)	6106 (26.8)	6021 (25.3)	5786 (23.8)	6073 (23.9)	6156 (23.2)	6231 (23.2)	6277 (22.5)
School or workplace			859 (3.8)	815 (3.6)	882 (3.7)	978 (4.0)	1082 (4.3)	1021 (3.9)	1117 (4.2)	1178 (4.2)
Hospital/private practice			690 (3.0)	693 (3.0)	767 (3.2)	840 (3.5)	1354 (5.3)	1816 (6.8)	1917 (7.1)	2105 (7.5)
Sport area			923 (4.1)	882 (3.9)	921 (3.9)	990 (4.1)	982 (3.9)	1105 (4.2)	1153 (4.3)	976 (3.5)
Other			497 (2.2)	550 (2.4)	638 (2.7)	495 (2.0)	443 (1.7)	491 (1.9)	419 (1.6)	450 (1.6)

PEMS: prehospital emergency medical services; SMUR: emergency physicians sent simultaneously onsite;

*The medical problem is related to the ECC keyword and not to a clinical diagnosis.

### Evolution of process indicators over time

The proportion of missing reports, assessable for SMUR interventions only, varied between 4.3% and 9.6% of all interventions over time, according to the SMUR team and region of activity. Ambulance interventions were cancelled after engagement in 3% of the cases, with a proportion who remain stable over years (2.5% in 2003 vs. 3.1% in 2010). The interventions time to start was stable during the study period for the SMUR with a mean < 5 minutes but slightly increased for the ambulances, especially since 2008 (Table 3). An increased trend of time to response was observed over years, especially for ambulances. It was mainly due to longer time for the travel to the scene. Mean duration on the scene was generally higher than 20 minutes for ambulances, except for paediatric patients (mean: 17 min), and was longer for people ≥ 80 years old. The mean duration of SMUR interventions (i.e. from call to availability for a new intervention) increased during the period of observation (38.5 to 43 min). For ambulance, mean duration of interventions also increased, but higher for elderly (+11 min) compared to adults (+7 min) or paediatric patients (+6 min).

**Table 3:** **Process indicators for SMUR interventions between 2000 and 2010**

Indicators	2001	2002	2003	2004	2005	2006	2007	2008	2009	2010
**Time to start (min)** ^**$**^										
Ambulance/SMUR	-/4(2)	-/4(3)	4(2)/4(3)	3(2)/4(3)	4(2)/4(3)	4(2)/4(3)	3(2)/4(3)	4(2)/4(3)	6(2)/4(3)	5(2)/4(3)
**% of departures > 5 min**										
Ambulance/SMUR	-/14.5	-/14.1	18.8/12.6	17.6/16.7	16.6/15.7	17.4/13.1	17.4/11.7	21.1/13.7	19.9/15.0	15.3/15.3
**Time to response (min)** ^**#**^										
Ambulance/SMUR	-/11(7)	-/11(7)	11(6)/11(7)	11(6)/11(7)	12(6)/11.5(7)	11(6)/11(7)	11(6)/11(7)	13(7)/12(8)	17(8)/12(8)	16(7)/12(8)
**Time on scene (min)** ^**£**^										
Ambulance										
*Age <16*			17(15)	18(16)	18(16)	18.5(16)	18(16)	17(15)	17(15)	17(15.5)
*Age 17-79*			20(19)	21(19)	21(19)	20(19)	20(19)	20(19)	20(19)	20(18)
*Age > =80*			22(20)	22(21)	22(21)	22(21)	23(21)	23(22)	23(22)	23(22)
SMUR										
*Age <16*	17(14)	15(13)	17(14)	19(16)	19(17)	17.5(16)	16.5(15)	17.5(15)	17(15)	18(15)
*Age 17-79*	20(19)	21(19)	21(19)	22(20)	22(20)	20(20)	20(19)	23(20)	23(21)	23(21)
*Age > =80*	20(19)	21(20)	20(19)	21.5(21)	21(21)	20(20)	20.5(21)	22(21)	22(21)	22(21)
**% of time on scene > 20 min**										
Ambulance/SMUR	-/42.3	-/45.0	51.5/43.7	53.0/50.5	53.5/47.7	52.4/59.3	51.4/58.3	52.1/48.9	51.8/51.2	51.9/50.3

Values are means (medians) unless indicated.

PEMS: prehospital emergency medical services; SMUR: emergency physicians sent simultaneously onsite; ^**$**^Time to departure: duration from alarm to start of the vehicle; ^**#**^Time to response: duration from alarm to arrival on scene; ^**£**^Time on scene: duration from arrival to leave from the scene.

### 48-hours patients’ outcome for PEMS interventions

The proportion of people alive and out of hospital after 48 h remained stable over years around 37% (Table 4). This proportion was higher among patients suffering from a psychiatric problem (70%). Around 3% of people alive were not brought to hospital, a proportion which remained constant over years. Overall survival after 48 h remained constant over years (87.6 to 89.2%). However, when related to specific pathologies, survival after 48 h regularly increased in case of cardiac arrests (+10.6% between 2001 and 2010) or myocardial infarction (+6.9% between 2001 and 2010).

**Table 4:** **48-hours outcomes of SMUR interventions between 2000 and 2010**

Indicators	2001	2002	2003	2004	2005	2006	2007	2008	2009	2010
**All interventions**	4879	5361	5652	5436	5581	5413	5303	4782	4991	5158
**Alive, not brought to hospital**	182(3.7)	164(3.1)	188(3.3)	171(3.1)	192(3.4)	183(3.4)	142(2.7)	143(3.0)	180(3.6)	165(3.2)
**Dead on the scene**	375(7.7)	405(7.8)	387(6.9)	385(7.1)	401(7.2)	395(7.3)	362(6.8)	361(7.6)	408(8.2)	391(7.6)
**Alive or Out of hospital at 48 h**	1844(37.8)	1932(36.0)	2069(36.6)	2108(38.8)	2016(36.1)	2059(38.0)	1928(36.3)	1710(35.7)	1962(39.3)	1958(37.9)
**Survival at 48 h**										
All interventions	4274(87.6)	4761(88.8)	5030(88.9)	4851(89.2)	4962(88.9)	4790(88.5)	4734(89.2)	4208(88.0)	4393(87.9)	4587(88.7)
Cardiac arrest	22(7.4)	31(9.7)	34(11.5)	29(10.2)	32(9.8)	28(8.5)	42(13.2)	56(16.1)	56(14.4)	77(18.0)
Myocardial infarction	199(88.4)	251(94.0)	218(91.2)	213(92.2)	245(88.5)	264(90.4)	298(92.8)	268(93.4)	308(93.1)	348(95.3)
Stroke	96(89.7)	111(89.5)	117(89.3)	99(90.8)	86(89.6)	97(89.0)	89(85.6)	76(82.6)	74(83.1)	182(92.4)
Sepsis	12(66.7)	20(71.4)	28(77.8)	22(73.3)	31(81.6)	24(72.7)	41(87.2)	41(83.7)	42(67.7)	48(84.2)

Values are numbers and percentages.

## Discussion

In this study, a 40% increase was observed in the number of requests to PEMS between 2001 and 2010 in a 700′000 inhabitant Swiss Canton. The overall rate of requests was 35 per 1000 inhabitants for ambulance services and 10 per 1000 for medical interventions (SMUR), with the highest rate among people aged 80 and above. Most frequent reasons for the intervention were related to medical problems, with a predominance of unconsciousness, chest pain, respiratory distress or cardiac arrest situations, whereas severe trauma interventions decreased over time. Overall, 89% were alive after 48 h.

In accordance with previous studies, these results confirm an increasing rate in PEMS requests and interventions [4, 19, 21]. Rate of request to PEMS was higher in the Canton of Vaud compared to Baden-Wuerttemberg, but the use of prehospital emergency services increased by year in the same proportion (16.2 to 19.9 per 1000 inhabitants per year between 2004 and 2008) [29]. The proportion of elderly patients requesting PEMS was comparable to other European studies [29]. The highest rate and the highest increase in PEMS requests and interventions are noticeable among people aged 80 and above, with a predominance of medical non–traumatic problems. These results are most likely related to demographic changes and to the gradual ageing of the Swiss population, leading to increasing PEMS interventions for elderly patients, especially those aged 80–89 [8, 30]. For these patients, situations related to the decline of general conditions or specific care impossible to be provided at home also increased, thus contributed to the observed evolution [31, 32]. These results are in accordance with previous publications and might indicate that new care pathways or supports need to be devised in the future for this specific population, in order to prevent overwhelming requests to PEMS and unnecessary transport to the hospital [33, 34]. Further investigations using a mixed qualitative and quantitative approach would probably provide policy options, to better predict the future development of the use of PEMS. The decrease in the number of primary care physicians and nursing home medical directors is another potential factor, influencing the rising rate of direct request to pre-hospital emergency medical services for these elderly patients [35].

Outcomes indicators could be measured for overall situations or specific medical pathologies. In terms of pathologies, overall injuries were decreasing, indicating possible changes in the epidemiology of trauma, particularly in the context of traffic accident prevention. The rates of myocardial infarction and their evolution over the time were similar with the Messelken study [29]. The mortality after myocardial infarction decreased regularly during the study period. The same was observed for cardiac arrest, but the success rate for cardiovascular resuscitation (CPR) was higher in our, possibly due to differences in definition or patients characteristics [29]. One explanation might be related to the optimization of the in-hospital treatment of those pathologies and therefore to their better prognosis. The evolution of the CPR guidelines and the implementation of national CPR campaigns may also have had an impact on the increase in survival after cardiac arrest. The general trend we observed will have to be confirmed in the following years; similar analyses are also needed in other regions or countries using the same PEMS organisation to assess the generalisability of those findings. A reduction in the number of patients who were not hospitalized at all was also perceptible. This might indicate a change in medical strategies on scene for less severe problems, or increased knowledge of the medical team for discriminating the severity of situations. This result goes in the direction of making a more appropriate management, with an indirectly cost-effective strategy. To assess the appropriateness of requests to PEMS for these situations, we might however perform a closer monitoring of quality indicators, to be defined, over years. Rates of cancelled missions stratified by age groups could for example help to quantify requests for "false" emergencies [6]. Nevertheless, our results contribute to emphasize the need to have an adequate and efficient health activity monitoring of the prehospital information system.

This study indicates that routine prospective collection of prehospital emergency interventions and monitoring of activity was feasible and successful over time in the Canton of Vaud. Even in the case of data collection through different databases, a picture of the activity over 10 years could have been drawn showing that the trends observed were similar than those previously reported. This was true for overall observations and taking account of the different PEMS existing in the World, and aforementioned restrictions for comparability of the systems. Documentation and reporting was larger for medical SMUR interventions, taking account of sets of variables described as fixed or optional [17, 25, 36]. This led to the possibility of measuring outcomes indicators for patients 48 h after the intervention. Quality of the reporting was very good for the set of ambulance data, and improved over years for SMUR datasets. Reporting rates were observed to be team and region dependent. Quality of data collection, however, improved generally over years which could be directly attributed to teams, as uniformity of data entry was assumed by a unique person moving in all intervention sites.

Our study and data collection have some limitations. The main was the non ability to link all three databases properly. The health information system must evolve to provide the information needed to measure, analyze, and understand the use of emergency services in order to improve their management and thus their effectiveness and efficiency. We will be able to address this limitation in the future as a uniform data collection is planned to be performed. Better matching of data would help to assess the appropriateness of use of ambulances and SMUR interventions. The evolution of the professions, trainings, diagnosis strategies and treatments are also important elements, contributing to the evolution and the improvement of PEMS performance. The respective contribution of each of these elements could not be evaluated in our study.

## Conclusion

A thorough analysis of existing and future data shall allow a better understanding of some determinants of the evolution of the use of emergency health services. More comprehensive analysis of the quality of services and patient safety supported by indicators are required. Coupled with additional assessments such as interviews or focus groups, they might help to develop pre-hospital emergency services and new processes of care.

## Abbreviations

PEMS: Pre-hospital emergency medical services
SMUR: Medical interventions
ECC: Emergency call center
DSS: Decision-support system
IUMSP: Institute of Social and Preventive Medicine.
